# A Cyclical Approach to Continuum Modeling: A Conceptual Model of Diabetic Foot Care

**DOI:** 10.3389/fpubh.2017.00337

**Published:** 2017-12-08

**Authors:** Martha L. Carvour, Allyssa Chiu

**Affiliations:** ^1^Division of Epidemiology, Biostatistics, and Preventive Medicine, Department of Internal Medicine, University of New Mexico, Albuquerque, NM, United States; ^2^Division of Infectious Diseases, Department of Internal Medicine, University of New Mexico, Albuquerque, NM, United States

**Keywords:** continuum model, cascade model, population health, diabetes, limb salvage, limb preservation, podiatry

## Abstract

“Cascade” or “continuum” models have been developed for a number of diseases and conditions. These models define the desired, successive steps in care for that disease or condition and depict the proportion of the population that has completed each step. These models may be used to compare care across subgroups or populations and to identify and evaluate interventions intended to improve outcomes on the population level. Previous cascade or continuum models have been limited by several factors. These models are best suited to processes with stepwise outcomes—such as screening, diagnosis, and treatment—with a single defined outcome (e.g., treatment or cure) for each member of the population. However, continuum modeling is not well developed for complex processes with non-sequential or recurring steps or those without singular outcomes. As shown here using the example of diabetic foot care, the concept of continuum modeling may be re-envisioned with a cyclical approach. Cyclical continuum modeling may permit incorporation of non-sequential and recurring steps into a single continuum, while recognizing the presence of multiple desirable outcomes within the population. Cyclical models may simultaneously represent the distribution of clinical severity and clinical resource use across a population, thereby extending the benefits of traditional continuum models to complex processes for which population-based monitoring is desired. The models may also support communication with other stakeholders in the process of care, including health care providers and patients.

## Introduction

Disease-specific “cascade” or “continuum” models (hereafter, collectively described as “continua”) have been developed for multiple diseases and conditions, including tuberculosis (1, 2), human immunodeficiency virus infection (3–5), hepatitis C virus infection (6–8), high blood pressure (9), and diabetes (10). Such models are used to define and measure important steps in care—such as screening, diagnosis, and treatment—on a population level.

Continua depict the desired, successive elements of care for a disease or condition, most often using bar graphs to represent the proportion of patients who receive care at each successive step in the continuum (1, 3, 6–11). These models can be used to identify gaps in care within a population or to compare the experiences of different subgroups or populations (3, 11–14) They can also inform the selection or evaluation of interventions intended to improve outcomes (11), including interventions that apply the principles of implementation science (15).

Recently, Perlman et al. set forth a series of constructive, methodological considerations that should be addressed in future continuum modeling, in order to optimize the utility of these models as population health tools (11). The authors advocate for continua that reflect the time elapsed between steps in the continuum; the incidence at each step (that is, not only the prevalence, as is measured by many traditional continua); the effect of preventive and therapeutic measures on continuum outcomes; the complex relationships between different conditions; and individual-level characteristics, such as migration, relapse, and death, that influence the population at risk for each successive step.

Here, we describe the development of a novel continuum model for diabetic foot pathologies—an example of a chronic and complex pathology for which continuum-type modeling may be valuable, yet for which the principles of traditional continuum modeling do not directly apply. Our proposed model may offer some preliminary advances toward the methodological considerations elucidated by Perlman et al. and suggest potential applications to other continua in the future.

## Diabetic Foot Pathologies

Diabetes mellitus is a prevalent chronic condition affecting over 29 million Americans (16, 17). Diabetic foot pathologies, including diabetic foot ulcers and foot infections, are a significant risk factor for disability, surgical amputation, and mortality among patients with diabetes (16, 18–20). Patients who undergo a diabetes-associated amputation have an estimated 5-year mortality rate over 50%, a rate exceeding that for many cancers (18, 20).

While diabetic foot pathologies represent a substantial health burden across the United States, the impact of these conditions varies disproportionately within the population. The prevalence of the underlying condition—diabetes mellitus—differs substantially across racial and ethnic groups, with a disproportionate impact among Hispanic, African-American, and American Indian/Alaskan Native populations (17). Meanwhile, the risk for amputation also differs across racial and ethnic groups (19, 21, 22), and these disparities may persist even when predisposing factors for amputation (such as infection) are comparable across groups (21).

Despite the population-wide impact of diabetic foot pathologies and the evidence for disparate outcomes within the population, there are currently no systematic epidemiological methods to define and measure the process of care for at-risk patients, including those who may be disproportionately affected by the condition or the associated risk of amputation. We propose that continuum modeling could help to address these gaps in our population-based understanding of diabetic foot disease, by measuring and depicting the burdens, outcomes, and disparities of these conditions across the population.

## Barriers to Complex Continuum Modeling

Recently, we set out to define a continuum of care for patients who are at risk for diabetic foot pathologies [A diabetes cascade published in 2014 emphasizes control of glucose, lipids, and blood pressure but does not extend to the evaluation and management of diabetic foot pathologies (10).]. In this process, we identified at least three aspects of diabetic foot care that do not fit traditional models, which would need to be addressed by our proposed model:

- The process for diabetic foot care is non-sequential. Unlike applications from some other continuum models which rely on stepwise sequences, such as screening, diagnosis, and treatment, the elements of care for patients at risk for diabetic foot pathologies do not have a consistently stepwise sequence. For instance, referral to Endocrinology or Podiatry specialists may be appropriate for patients who are at risk for diabetic foot pathologies, and these referrals may be viewed as important elements of care. However, these cannot be arranged in a standard hierarchy for an entire population of patients with diabetes.
- Some elements of care are repeated. Patients who are at risk for diabetic foot pathologies should have access to ongoing diabetes management, even if those patients have already developed advanced foot pathologies. For instance, a patient with a serious foot infection should receive ongoing glucose control measures as well as preventive screening of the unaffected extremity. This differs from some traditional continuum models, in which successive steps in care are deemed complete if they occur once.
- The desired endpoints of care are complex. In traditional continuum models, the desired endpoint is often a well-defined clinical outcome, such as a clinical cure. By contrast, a single, measurable endpoint cannot be defined for all patients at risk for diabetic foot pathologies. For instance, limb preservation (or avoidance of surgical amputation) may only be viewed as a desirable outcome in patients with advanced disease but not those with uncomplicated diabetes.

**Figure 1:**
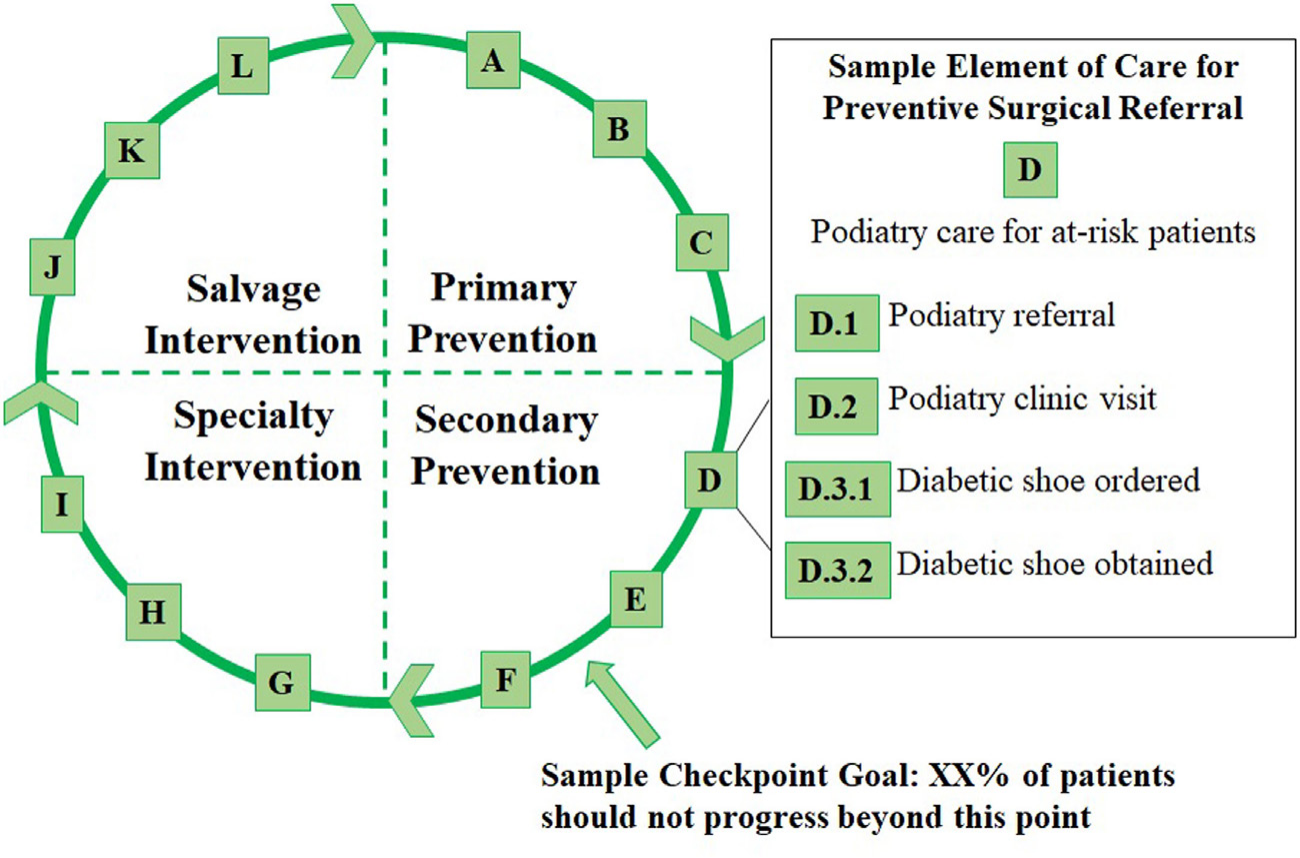
Overview of the Cycle with four phases of care, a sample checkpoint, and a sample element of care involving preventive podiatric referral.

To address these factors, we considered extending other models, such as the existing cascade for diabetes (10), as well as models which apply a limited number of branch points along a traditional sequential cascade (9, 23). However, we propose that a traditional continuum model, with sequential elements appearing along a single axis (e.g., as depicted on a traditional bar graph) or along an axis with limited branch points, cannot adequately capture the complex, desired elements for diabetic foot care. In response to this challenge, we devised an innovative model for diabetic foot pathologies that is configured as a continuous *cycle* of care (the “Cycle”). Figure 1 provides a simplified conceptual overview of the Cycle.

## Composition of the Cycle

The Cycle is divided into *four phases* representing the prevention and treatment of diabetic foot pathologies. Each phase represents a graduated risk for surgical amputation. These are defined as follows:

- The first phase is “Primary Prevention,” which applies to all patients with diabetes. Patients in this phase should all receive primary care for glycemic control as well as standard preventive screenings (e.g., serial foot screening).
- “Secondary Prevention” includes patients who are at high risk for foot pathologies, based on existing clinical criteria (24), or who have an early podiatric complication of diabetes (e.g., an uncomplicated foot ulcer).
- “Specialty Intervention” includes patients with one or more complicated foot pathology (e.g., a complicated foot ulcer or a superficially infected ulcer).
- “Salvage Intervention” includes patients with severe foot pathologies (e.g., infection of the bone or severe systemic infection) who require life- or limb-preserving treatments.

Each phase is further subdivided into clinically meaningful, evidence-based, and measurable *elements of care* that patients may receive in that phase or any successive phase. For instance, patients in the Specialty Intervention category should continue to receive the preventive care outlined in the two earlier phases. The elements are arranged using letters instead of numbers to signify that, although various elements can be placed within clinically discernable phases, elements within each phase may not follow a defined sequence (e.g., D includes Podiatry referral while E includes Endocrinology referral).

We have defined elements A through L along with a series of sub-elements (e.g., D.1, Figure 1) in each lettered category. The elements within each phase of the Cycle are summarized below, and examples of sub-elements in each category are given.

Primary Prevention includes:

A: Diabetes primary care, e.g., diagnosis and type of diabetes and visits to a primary care provider.
B: Glucose primary care, e.g., prescription of hypoglycemic agents and performance of glucose monitoring.
C: Foot primary care, e.g., completion of serial foot screening examinations by a primary care provider.

Secondary Prevention includes the following:

D: Podiatry care for patients at high risk for foot pathologies, e.g., podiatry referrals, podiatry visits, and diabetic shoe prescriptions.
E: Endocrinology care for patients at high risk for foot pathologies, e.g., endocrinology referrals, endocrinology visits, prescription of hypoglycemic agents, and performance of glucose monitoring measurements.
F: Podiatry care for uncomplicated foot ulceration, e.g., diagnosis and staging of ulcers, debridement of ulcers, and prescriptions for an offloading device or dressing.

Specialty Intervention includes the following:

G: Podiatry care for complicated foot ulceration without evidence of infection; e.g., diagnosis and staging of ulcers; debridement of ulcers; application of an offloading device, dressing, or procedure; and referral to another specialty (such as Vascular Surgery).
H: Interventions for superficial diabetic foot infection, e.g., diagnosis and type of infection; debridement procedures; application of an offloading device, dressing, or procedure; referral to another specialty (such as Vascular Surgery); and antibiotic prescriptions.
I: Interventions for a deep diabetic foot infection, e.g., diagnosis and type of infection; radiological imaging; microbiological assessment; debridement procedures; referral to another specialty (such as Infectious Diseases); and antibiotic prescriptions.

Salvage Intervention includes:

J: Interventions for diabetic foot infections involving the joints and/or bones in the foot or leg, e.g., diagnosis and type of infection, radiological imaging, microbiological assessment, type of procedure (such as incision and drainage), referral to another specialty (such as Infectious Diseases or Orthopedic Surgery), and antibiotic prescriptions.
K: Interventions for diabetic foot infections complicated by sepsis and other severe complications, e.g., diagnosis and severity of sepsis or other complications, type of supportive care (such as hospitalization, vasopressor support, or antibiotics), and associated procedures (such as central venous catheter placement and arterial line placement).
L: Limb amputations involving any portion of the leg, foot, or toes, e.g., indication for amputation and type of procedure.

## Interpretation of the Cycle

A patient enters the Cycle in the phase corresponding to their first diabetes-related diagnosis. For instance, patients with diabetes without foot pathologies enter the Cycle at Primary Prevention, whereas those with one or more uncomplicated foot ulcers enter the Cycle at Secondary Prevention. Patients remain in the Cycle indefinitely, although they may move to earlier or later phases over time or skip phases (e.g., if their condition rapidly accelerates or if their condition rapidly improves after treatment). Similarly, patients who progress through the Cycle to a surgical amputation remain in the Cycle for ongoing preventive care involving the intact portion of the affected extremity or the other lower extremity. This emphasizes the continuous and cyclical nature of diabetic foot care.

The goal for each *individual* in the population is to slow or stop clinical progression to later phases in the cycle. Meanwhile, the goal for the *population* is to reduce the proportion of patients who progress beyond certain checkpoints (see Figure 1) to more advanced phases of the cycle. By tracking patients’ individual experiences within the Cycle and aggregating this information on a population level, experiences across the population can be measured and a cyclical continuum model can be applied.

## The Cyclical Continuum

Cyclical continuum modeling may allow population health researchers to adapt the principles of traditional continuum modeling to a complex clinical entity, such as diabetic foot pathologies, with a significant population health impact. By superimposing circular graphical methods on the Cycle (e.g., simple circular charts for all users or complex circular visualization tools for advanced users), researchers can depict the proportion of the population engaged in various phases and elements of diabetic foot care as well as the distribution of health services or health spending across the phases. These distributions can be compared over time (e.g., following an intervention intended to reduce amputation rates) or across subgroups within the population where disparities may occur.

**Figure 2:**
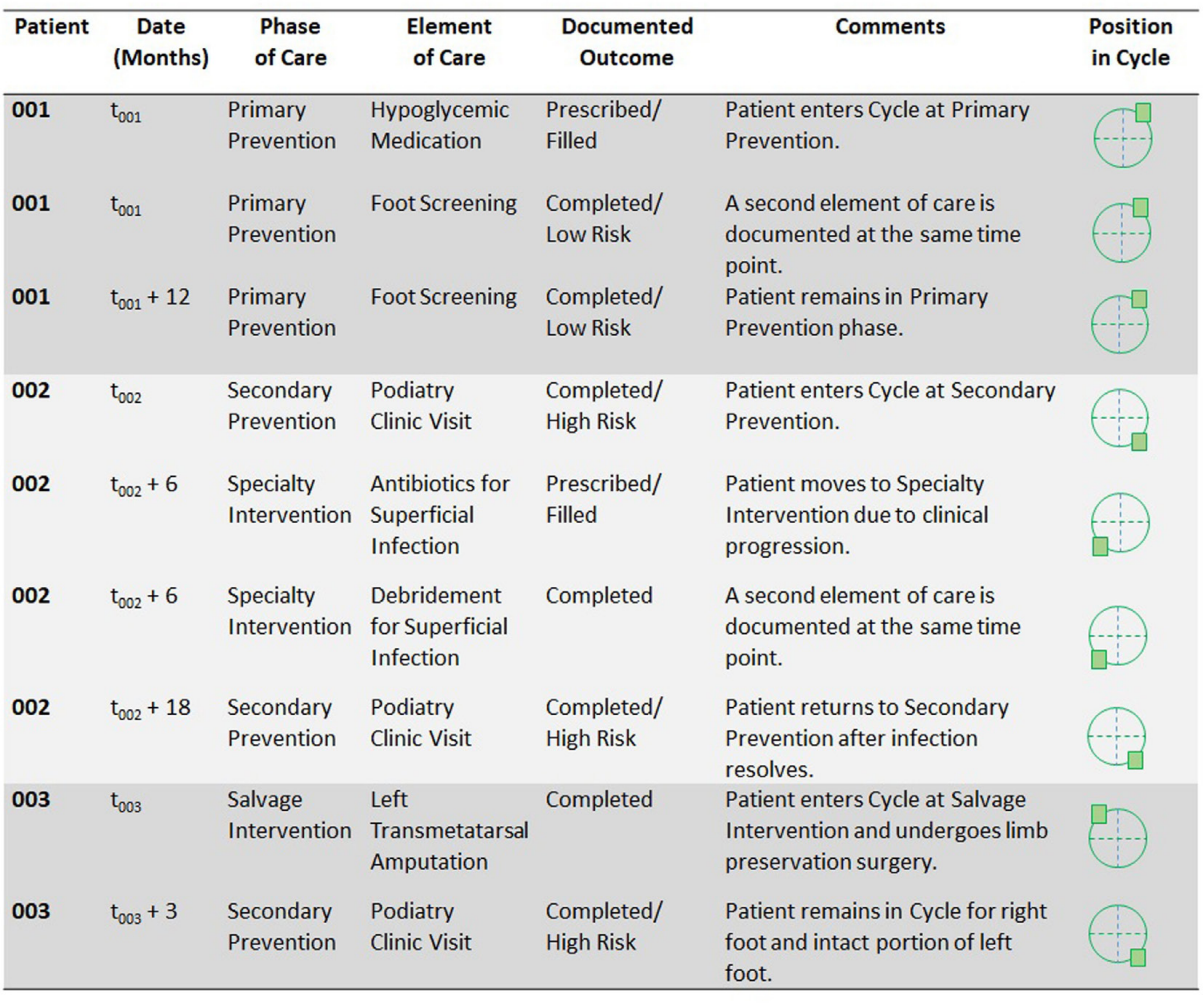
Proposed illustration of individual-level data that could be recorded using a Cycle model and that could support incidence calculations and time-to-event analyzes.

Importantly, cyclical continuum modeling may address several methodological obstacles that are inherent in traditional models. Cyclical modeling reflects the natural complexity of care for many clinical conditions, by permitting a set of interrelated endpoints to exist in the same model. It recognizes that important elements of care may not fall along a stepwise, non-repeating sequence, yet it recognizes the organization of these elements within successive clinical phases. Furthermore, it emphasizes the individual experience of each patient—who may move between phases or even repeat the cycle—thereby permitting detailed measurement of the individual-level experience, including preventive care, treatments, and outcomes over time. Collection of individual-level data for a cyclical model (see illustration in Figure 2) may also permit incidence-based and time-to-event calculations.

## Illustrations of the Cycle

To illustrate potential applications of the Cycle, Figure 2 summarizes three patient scenarios. Each patient receives a unique identifier to permit data linkage over time (and ideally, linkage from one data collection site to another). Time points are then recorded for all initial and subsequent data entries, so that incidence-based and time-to-event calculations can later be performed.

Patient 001 enters the Cycle at the Primary Prevention phase. He carries a diagnosis of diabetes without high risk for diabetic foot pathologies. During the patient’s first encounter recorded in the database, he receives a prescription for a hypoglycemic medication (element B) and undergoes a foot screening examination (element C). When patient 001’s data are later incorporated into population-level calculations, he would be included in the count (or numerator) for each of the elements recorded as well as the count (or denominator) for both the Primary Prevention phase and the Cycle as a whole. One year later, at *t*_001_ + 12 months, the patient has another encounter for a foot screening examination. The Cycle database captures not only the occurrence and timing of this event, but its recurrence in the care process (e.g., according to screening guidelines).

Patient 002 enters the Cycle at the Secondary Prevention phase. She has diabetes with high risk for foot pathologies. She is referred to a podiatrist (element D.1) and undergoes a podiatric examination (element D.2). At a visit 6 months later, she is diagnosed with a superficial infection for which antibiotics are prescribed and debridement is performed, so she advances in the Cycle to the Specialty Intervention phase. At a subsequent encounter, she undergoes a regular podiatry examination, and she has no evidence of recurrent infection at that time. She returns to the Secondary Prevention phase. Throughout her time in the Cycle, ongoing Primary Prevention (such as primary care visits) and Secondary Prevention (such as an Endocrinology visit that occurs when the patient is still in the Specialty Intervention phase) may still be recorded. As the patient moves between phases, the time elapsed between these can be captured for incidence-based and time-to-event calculations on the population level.

Patient 003 enters the Cycle at the Salvage Intervention phase. His first encounter involves a transmetatarsal amputation of the left foot (element L) for a severe diabetic foot pathology. If his right foot had no evident pathologies, he would still enter the Cycle at the most advanced stage for which he qualified—that is, at Salvage Intervention, because of the condition of his left foot. Following the amputation procedure, he remains in the Cycle for the care of his right foot and the intact portion of his left foot. If both of these extremities remain free of pathology, he would return to the Secondary Prevention phase (diabetes with high risk for foot pathologies). Thus, the Cycle permits tracking of multiple extremities per patient and allows for ongoing follow-up of patients who have undergone an amputation procedure.

## Potential Clinical and Translational Applications

Lastly, we propose that a cyclical continuum that emphasizes the experience of each individual within the population and that reflects the natural complexities inherent in that patient’s care may offer another advantage—that is, direct applications as a clinical communication tool among patients and providers, each of whom represents an important stakeholder in the process of care on the population level. The phases of care in the Cycle reflect the importance of interdisciplinary medical and surgical care for patients at risk for diabetic foot pathologies. Meanwhile, the Cycle reinforces the significance of maintaining primary and secondary preventive care in all patients with diabetes, including those with progressive or complicated diabetic foot pathologies.

For health care *providers* then, the Cycle may support effective collaboration with other providers—such as timely referral for preventive surgical care (e.g., D: Podiatry care for at-risk patients, Figure 1)—by depicting appropriate circumstances for referral and by providing a common basis for communicating clinical assessments. For *patients*, the tool may visually link preventive measures with avoidable outcomes, such as surgical amputation. This may be particularly valuable, since evidence elsewhere suggests that a patient’s conceptualization of this link may influence his/her likelihood of following preventive foot care practices (25, 26). For *population health researchers*, the simultaneous use of this tool in the clinical and research environments may improve communication with clinical stakeholders and foster the implementation of population-based interventions intended to improve outcomes.

## Conclusion

In summary, the Cycle represents a novel approach to continuum modeling for complex clinical conditions. This model accommodates the non-uniform and non-sequential steps needed to prevent and treat diabetic foot pathologies, while still allowing for population-based assessments of access to care, outcomes of care, and potential disparities of care within the population. By preserving a focus on the individual’s experience within the Cycle, the tool may permit more robust continuum modeling, including incidence calculations and time-to-event analysis, in the future. It may also have desirable clinical applications for communicating with health care providers who work with patients at different phases of the Cycle and for patients who are at risk for diabetic foot complications.

Here, the Cycle is illustrated as a conceptual model. Future work should extend the conceptual basis for cyclical continuum modeling among patients at risk for diabetic foot pathologies, by developing replicable methods for tracking each individual patient’s experience, quantifying the process of care (or barriers to care) across the population and within subgroups at risk for disparate outcomes, and applying circular visualization tools to the Cycle. Meanwhile, attempts to incorporate the Cycle into clinical practice as a communication tool for patients and providers should be considered, and the qualitative and quantitative impacts of the tool on clinical communication and patient outcomes should be measured.

## Ethics Statement

This work describes a methodological framework for epidemiological research. No research data involving human subjects are reported.

## Author Contributions

MC and AC were involved in the conception and design of this work and in the preparation and revision of the manuscript. MC and AC approved the final version of the manuscript and agreed to be accountable for its content.

## Conflict of Interest Statement

The authors declare that the research was conducted in the absence of any commercial or financial relationships that could be construed as a potential conflict of interest.
